# Using RMG Out-of-the-Box for Formic Acid Pyrolysis and Oxidation

**DOI:** 10.1021/acsomega.5c11182

**Published:** 2026-01-15

**Authors:** Jintao Wu, Alon Grinberg Dana

**Affiliations:** † Grand Technion Energy Program (GTEP), Technion—Israel Institute of Technology, Haifa 3200003, Israel; ‡ Wolfson Department of Chemical Engineering, Technion—Israel Institute of Technology, Haifa 3200003, Israel; § Boeing-Technion SAF Innovation Center, Technion—Israel Institute of Technology, Haifa 3200003, Israel

## Abstract

The predictive modeling of formic acid (HOCHO), the simplest organic acid and a central intermediate in combustion chemistry, is of fundamental importance. Prior literature work [*Energy Fuels* **2022,** *36,* 23, 14,382–14,392] reported challenges in reproducing jet-stirred reactor (JSR) speciation with an automatically generated model, suggesting that hand-tuned mechanisms might be required. Here, we revisit this assessment. We demonstrate that an out-of-the-box, fully automated predictive model, built deliberately without quantum-chemical refinement or ad hoc fitting, reliably captures formic acid JSR oxidation speciation across 550–1150 K and an equivalence ratio range of 0.5–2.0 as well as laminar burning velocity observations. We further reveal that the model’s remaining discrepancies, which appear under pyrolysis conditions, stem from a core issue masked in prior work [*Combust. Flame* **2021,** *223,* 77–87]. Specifically, we show that previous apparent agreement relied on fitted pressure-**in**dependent rate coefficients that obscured the unresolved pressure-dependent branching ratio between decarboxylation (HOCHO ⇌ CO_2_ + H_2_) and dehydration (HOCHO ⇌ CO + H_2_O). This case underscores that genuine scientific advancement requires the transparent discussion of successes alongside remaining challenges rather than masking theoretical discrepancies through parameter fitting or using selective benchmarks. By sacrificing perfect agreement in favor of highlighting true challenges, we establish that predictive, automated chemical kinetic models are already within reach for small oxygenated fuels, and identify the accurate parameterization of pressure-dependent rate coefficients on the CH_2_O_2_ surface as the key remaining challenge for fully reliable formic acid modeling.

## Graphical abstract

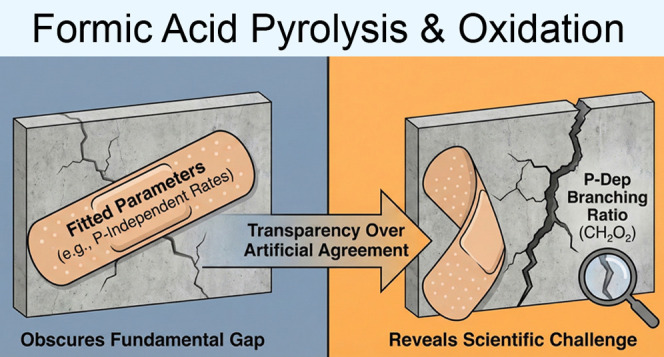

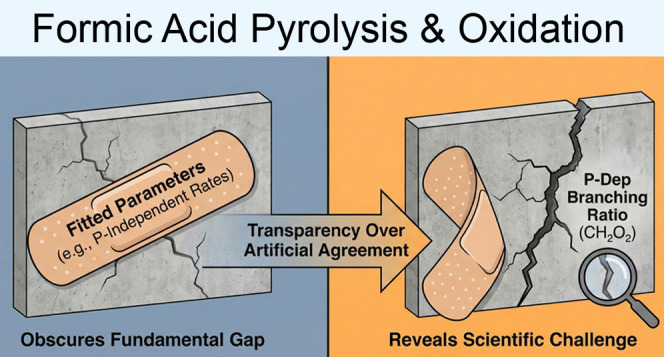

## 1 Introduction

Formic acid (HOCHO), systematically named methanoic acid, is the simplest carboxylic acid and a central intermediate in the oxidation of larger oxygenated biofuels.^1,2^ Due to the absence of carbon–carbon bonds, formic acid has been explored as a diesel fuel additive capable of lowering NO_*x*_ and particulate emissions.^3^ Beyond this application, it is considered a promising candidate for hydrogen storage and direct fuel use.^4^ A comprehensive understanding of the pyrolysis and oxidation kinetics of formic acid is, therefore, essential to reveal the core mechanisms governing the combustion of oxygenated hydrocarbons. Despite this relevance, knowledge of its gas-phase oxidation chemistry remains incomplete.^5^

Marshall and Glarborg^6^ developed a chemical kinetic model for the oxidation of formic acid, including calculated rate coefficients for hydrogen abstraction reactions by key radicals. They validated their model against laminar burning velocity measurements reported by de Wilde and van Tiggelen.^7^ Building on this foundation, Yin et al.^8^ extended the Marshall and Glarborg mechanism by incorporating additional reactions derived from ab initio calculations. Their updated model was benchmarked, alongside the original Marshall and Glarborg mechanism,^6^ against newly acquired jet-stirred reactor (JSR) speciation data covering both pyrolysis and oxidation conditions.

To improve model–experiment agreement, Yin et al. fitted rate coefficients for several key reactions, including those from the H_2_/O_2_ subset, hydrogen abstraction reactions, and the unimolecular decomposition pathways of formic acid into CO + H_2_O and CO_2_ + H_2_.^8^ Notably, although they computed pressure-dependent rate coefficients for the unimolecular decomposition of formic acid (Figures 7 and 9 in their work^8^), their model employed high-pressure-limit rate coefficients for these decomposition reactions. For the HOCHO ⇌ CO + H_2_O reaction (Reaction R1), Yin et al. took the high pressure limit rate coefficient from Marshall and Glarborg,^6^ while the rate coefficient of the HOCHO ⇌ CO_2_ + H_2_ reaction (Reaction R2) was manually fitted to agree with the experimental data. Furthermore, although Yin et al. reported a calculated rate coefficient for the reaction HOCHO + OH ⇌ HOCO + H_2_O,^8^ they ultimately used an earlier literature value in their published model.^9,10^ The adopted rate coefficient is approximately 4 orders of magnitude higher at 1000 K than their own computed value.^8^

A very recent study by Blitz et al.^11^ further clarified the kinetics of the HOCHO + OH reaction. Their high-level calculations are in good agreement with the rate coefficients derived by Anglada.^9^ Importantly, Blitz et al. demonstrated that the additional H atom forming channel proposed by Yin et al.^8^ to explain high-temperature reactivity possesses a barrier significantly higher than originally estimated (37 kJ mol^–1^ vs 22.6 kJ mol^–1^), effectively precluding it as a major source of H-radicals. Instead, tunneling-facilitated decomposition of HOCO was identified as the likely source of atomic hydrogen under these conditions.^11^

Recently, Wako et al.^12^ developed two chemical kinetic models for the oxidation of formic acid. The first model, designated as *KiBo_AG* in their work, was automatically generated using the Reaction Mechanism Generator (RMG) software.^13,14^ The second model, *KiBo_MU*, was manually updated by extending a prior light alkene oxidation mechanism.^15^ The latter incorporated a manually curated subset of reactions relevant to formic acid chemistry from the literature. In addition, several rate coefficients were manually adjusted to improve alignment with the experimental data.^12^ Both models were benchmarked against experimental measurements, including laminar burning velocities and JSR oxidation speciation data, using the Marshall and Glarborg mechanism^6^ and the updated AramcoMech2.0^5^ as reference models. However, the benchmarking set used in that work^12^ was selective: it excluded portions of the available experimental temperature for oxidation and did not include the available JSR data for pyrolysis, an important and relevant regime for formic acid combustion that is also more challenging to predict.

The automatically generated model, *KiBo_AG*, was reported to exhibit noticeable discrepancies with the experimental results under the conditions tested. In contrast, the manually updated model was shown to achieve better overall agreement with the species analyzed. The authors concluded that “Overall, *KiBo_MU* showed better agreement for all species; however, *KiBo_AG* showed some variation, especially for CO_2_ and H_2_.”^12^

The present work re-examines these conclusions by targeting an open question in formic acid oxidation modeling: Can a genuinely predictive—rather than postdictive^16^ or heavily parameter-fitted—chemical kinetic mechanism be generated for formic acid pyrolysis and oxidation using an “out-of-the-box” automated approach?

## 2 Methods

The chemical kinetic model developed in this work was automatically generated using the Reaction Mechanism Generator (RMG) software^13,14^ version 3.3.0. RMG explores possible intermediate species and associated elementary or pressure-dependent (well-skipping) reactions for a specified reacting mixture by using a combination of template-based reaction rules and precompiled libraries. Given user-defined initial conditions (mainly temperature, pressure, and reactant composition), RMG discovers the main chemical pathways and estimates unknown reaction rate coefficients and species thermochemistry using reaction family based decision trees and the group additivity method, respectively.^14,17^

Species and reactions are selected via a flux-based algorithm that ranks species importance according to a user-defined tolerance threshold. Pressure-dependent reaction networks were automatically modeled using a master equation (ME) describing molecular processes on a collisional time scale.^18^ The modified strong collision (MSC) approximation^19^ was used to solve the ME, as implemented in Arkane.^20^ Additional details on the underlying algorithms and capabilities of RMG can be found in the literature^13,21^ and in the official documentation.^22^

In this study, the chemical kinetic model was generated by defining a reactor system that encompassed the following conditions: temperature and pressure ranges of 450–2000 K and 1–50 bar, respectively, considering both oxidation conditions (equivalence ratio, ϕ, of 0.5–2.0) and pyrolysis conditions (a mixture of 2% formic acid in He). The tolerance parameter for species selection was set to 0.001, and a termination time of 5 s was used as the convergence criterion for all simulations. A summary of the RMG input parameters and a comparison to the parameters used by the automated model reported by Wako et al.^12^ is given in Table S1. To assess the generalizability of the approach and capture cross-term kinetics, a supplementary model for mixtures of formic acid with hydrogen (H_2_) and methane (CH_4_) was generated using the same methodology. Model simulations were performed using Cantera.^23^ In laminar burning velocity computations, the Soret effect and multicomponent transport were first neglected to generate an initial estimate and later incorporated in the final solution for improved accuracy.

In this work, we employ RMG in an “out-of-the-box” manner, which we define as generating a mechanism using standard, publicly available thermochemical and kinetic libraries without subsequent quantum chemical refinement or ad hoc parameter fitting. This resulting “Vanilla” model represents the baseline predictive capability of the current automated framework rather than a tuned or hybrid derived model.

## 3 Results and Discussion

Figure 1 reproduces a key modeling benchmark from Wako et al.,^12^ where an automatically generated model (*KiBo_AG*) was found to significantly underpredict experimental species profiles for formic acid oxidation compared to a manually updated (tuned) version (*KiBo_MU*). The automatically generated model was shown to significantly underpredict the mole fractions of CO_2_, CO, and H_2_ against JSR oxidation data under stoichiometric conditions.

**Figure 1:**
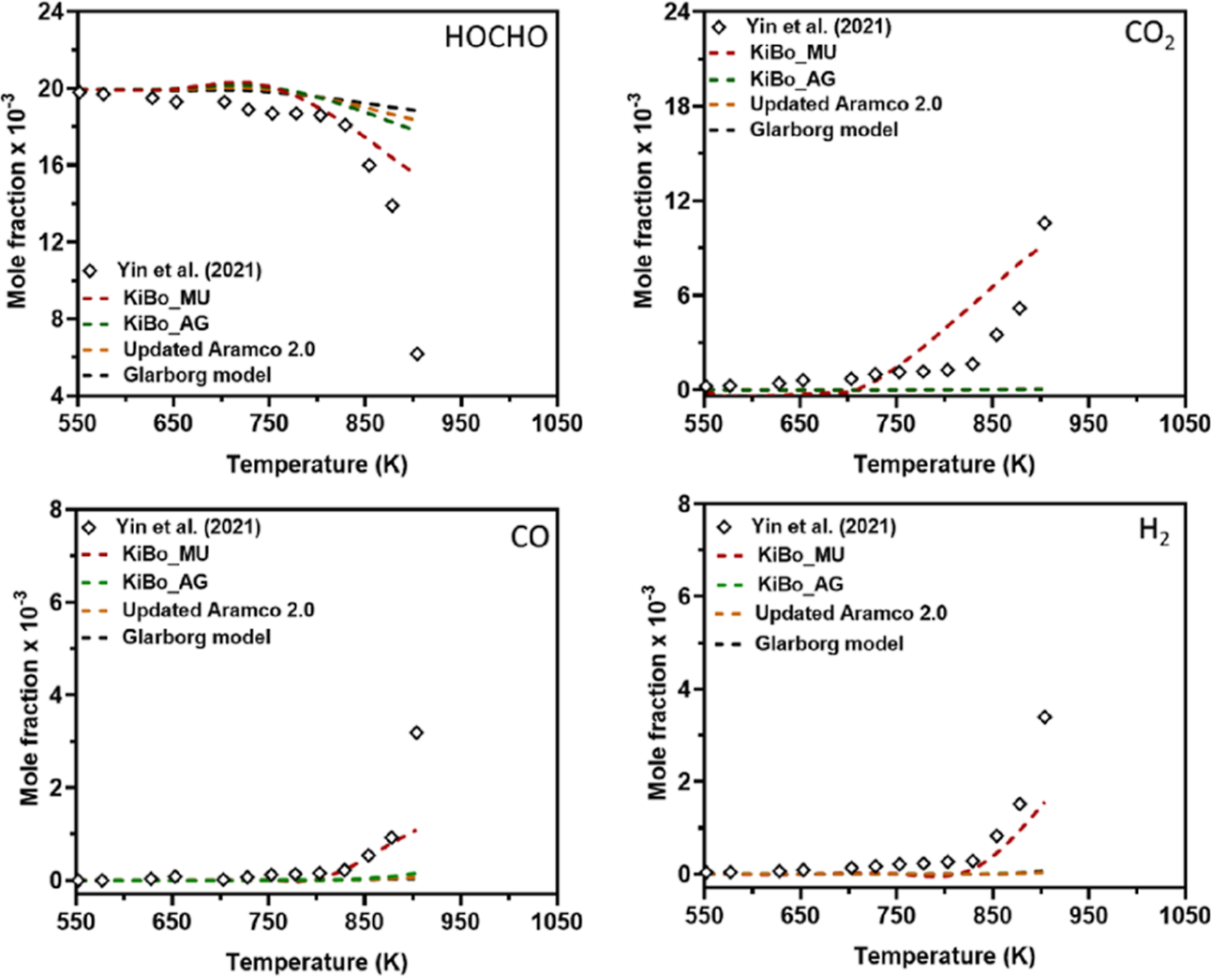
A literature report of mole fraction profiles of HOCHO, CO_2_, CO, and H_2_ at temperatures of 550–900 K, an equivalence ratio of 1.0, atmospheric pressure, and a residence time of 2 s in a JSR. Symbols: experimental data from ref 8; broken lines: simulation results from ref 12. This figure was reproduced from ref 12: *Energy Fuels* **2022,** *36,* 14,382–14,392, F.M. Wako, G. Pio, E. Salzano, Modeling Formic Acid Combustion, Copyright American Chemical Society (2022).

The benchmark by Wako et al.^12^ relied on a significantly truncated experimental validation set, terminating the comparison at ∼900 K for ϕ = 1.0. This restriction excluded the critical high-temperature regime available from the same experimental data set (up to 1000–1050 K, depending on the species), where experimental species profiles exhibit marked shifts in reactivity trends (see the full ϕ = 1.0 JSR data series in Figure 3 below). Furthermore, the analysis entirely omitted the available JSR pyrolysis data, which is important for combustion conditions as well, available from the same experimental source.^8^ Their analysis therefore avoided the most rigorous test of the mechanism’s validity, thereby resulting in an incomplete assessment of model performance. This pyrolysis regime is known to pose a substantially greater challenge for kinetic modeling, as discussed below.

**Figure 2:**
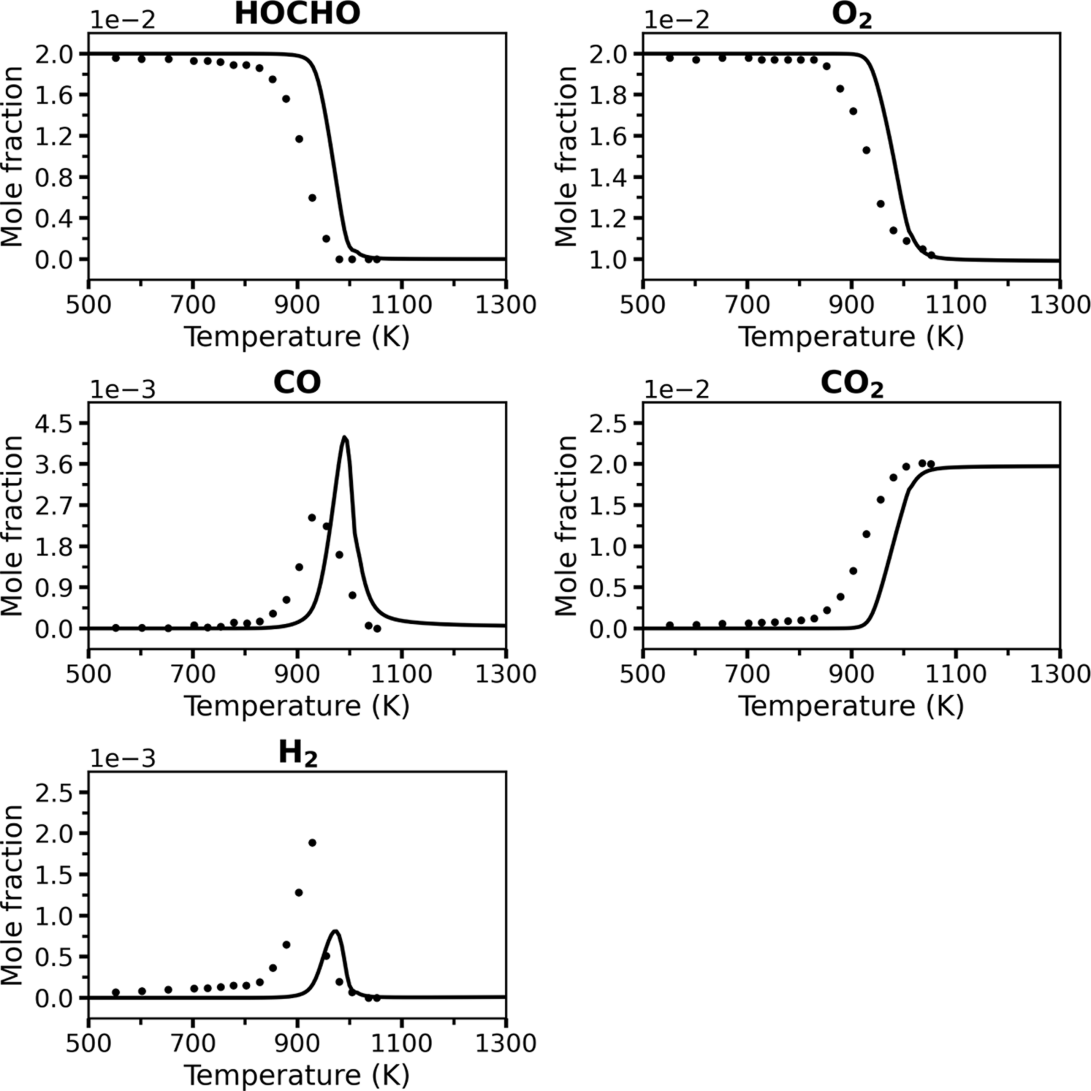
A comparison of the present “Vanilla” RMG model (solid lines) and JSR experimental data^8^ (symbols) for key species at 1 atm and ϕ = 0.5, with a 2 s residence time.

**Figure 3:**
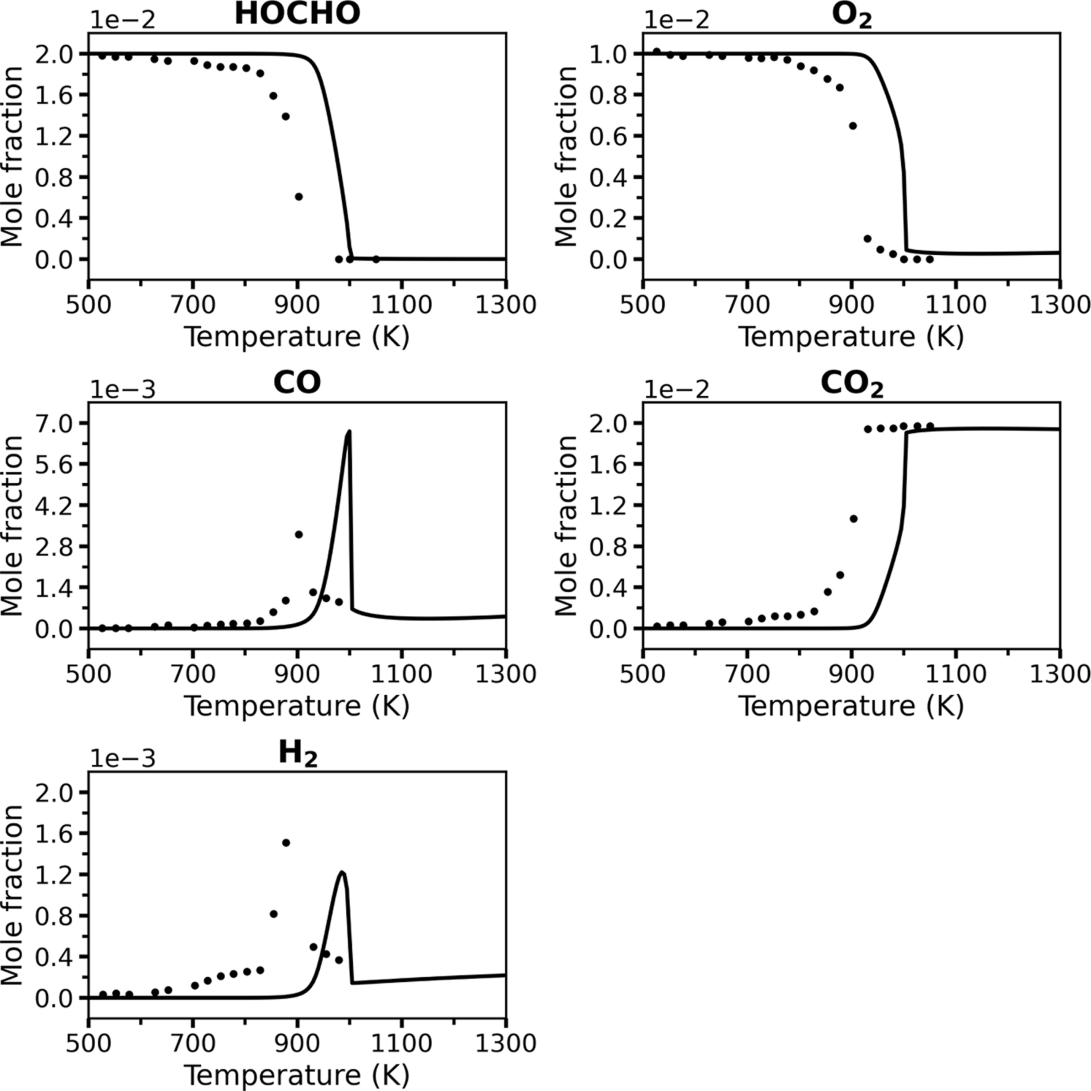
A comparison of the present “Vanilla” RMG model (solid lines) and JSR experimental data^8^ (symbols) for key species at 1 atm and ϕ = 1.0, with a 2 s residence time.

Figures 2–4 compare the predicted mole fractions of key species from the RMG model generated in the present work and the JSR experimental data (that have a reported estimated uncertainty of ±10% in mole fraction^8^) under oxidation conditions at 1 atm with equivalence ratios of ϕ = 0.5, ϕ = 1.0, and ϕ = 2.0, each with a 2 s residence time. The present RMG model is termed “Vanilla” to emphasize that this is an “out-of-the-box” model with no quantum chemical refinement. For further insight into the controlling reactions under oxidation conditions, sensitivity analyses for the ϕ = 0.5 case are provided in the Supporting Information, Figures S1–S4.

**Figure 4:**
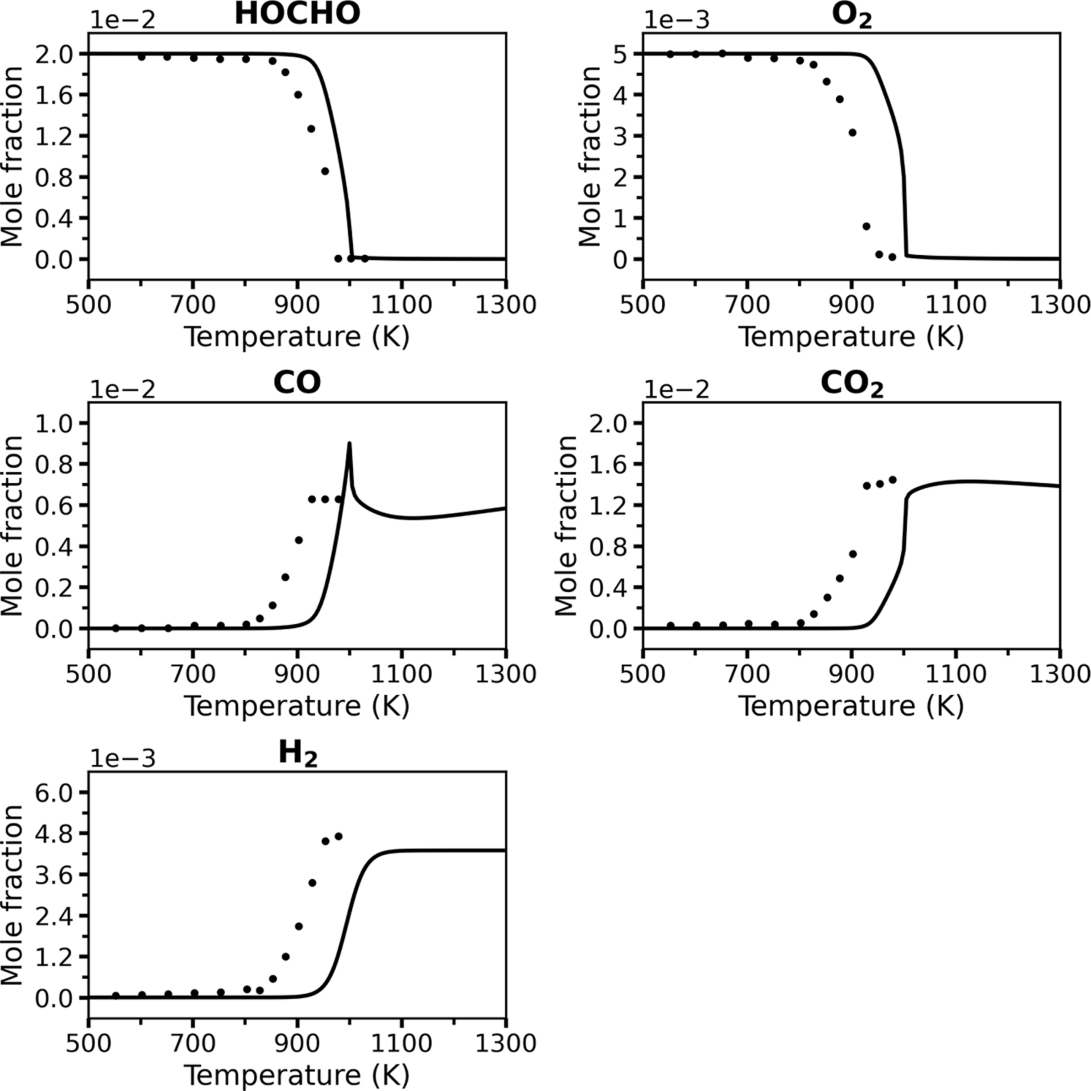
A comparison of the present “Vanilla” RMG model (solid lines) and JSR experimental data^8^ (symbols) for key species at 1 atm and ϕ = 2.0, with a 2 s residence time.

To evaluate the performance of the “Vanilla” model, it is important to define the criteria for predictive success in the context of automated mechanism generation. Unlike previous studies that utilized both quantum chemical calculations and parameter fitting to achieve near-perfect overlap with specific data sets,^8^ our goal is to assess the baseline fidelity of an unfitted, unrefined, first-principles model. This is an important stepping stone toward the next stage of refining the model, remaining physically based without parameter fitting. We consider a predictive yet unrefined model, such as the one reported herein, to reliably capture the chemistry if it: (1) reproduces the correct qualitative trends and species profiles as observed experimentally, in contrast to the negligible reactivity observed in prior automated attempts^12^ and (2) achieves quantitative agreement within a reasonable margin, specifically capturing the temperatures of oxidation onset and peak species concentrations within ∼50 K without ad hoc tuning.

Across all equivalence ratios, the “Vanilla” model successfully captures the qualitative and quantitative trends, as well as the overall reactivity of the system. The predicted profiles for the reactants, HOCHO and O_2_, and the main oxidation product CO_2_ follow the experimental data adequately well. While the model correctly predicts the characteristic peaks for the CO and H_2_ intermediates, their magnitudes show minor deviations, and a small artifact is observed for CO at rich fuel conditions. A consistent, slight shift of the reactivity to higher temperatures is observed across all conditions, suggesting the model reproduces the dominant reaction pathways correctly but may have minor inaccuracies in the effective activation barriers of key reactions. Nevertheless, the agreement across this wide range of conditions for an unrefined, untuned model is remarkable and demonstrates that a standard, automatically generated mechanism can provide a coherent and predictive description of formic acid oxidation. The “Vanilla” model demonstrates strong performance for an “out-of-the-box” RMG model.

Figure 5 presents a comparison of the predicted mole fractions of key species from the RMG “Vanilla” model and experimental JSR data^8^ under pyrolysis conditions at 1 atm and a 2 s residence time. The model reasonably captures the decomposition of formic acid with predicted HOCHO mole fraction profiles matching relatively well the experimental data across the entire temperature range. However, significant discrepancies are apparent for CO, CO_2_, and H_2_. For CO, the model underpredicted the peak mole fraction observed in the experimental data between 1050 and 1200 K by ∼30%. Furthermore, while the experimental CO_2_ and H_2_ profiles exhibit peaks, the model predicts broader curves in the relevant temperature range.

**Figure 5:**
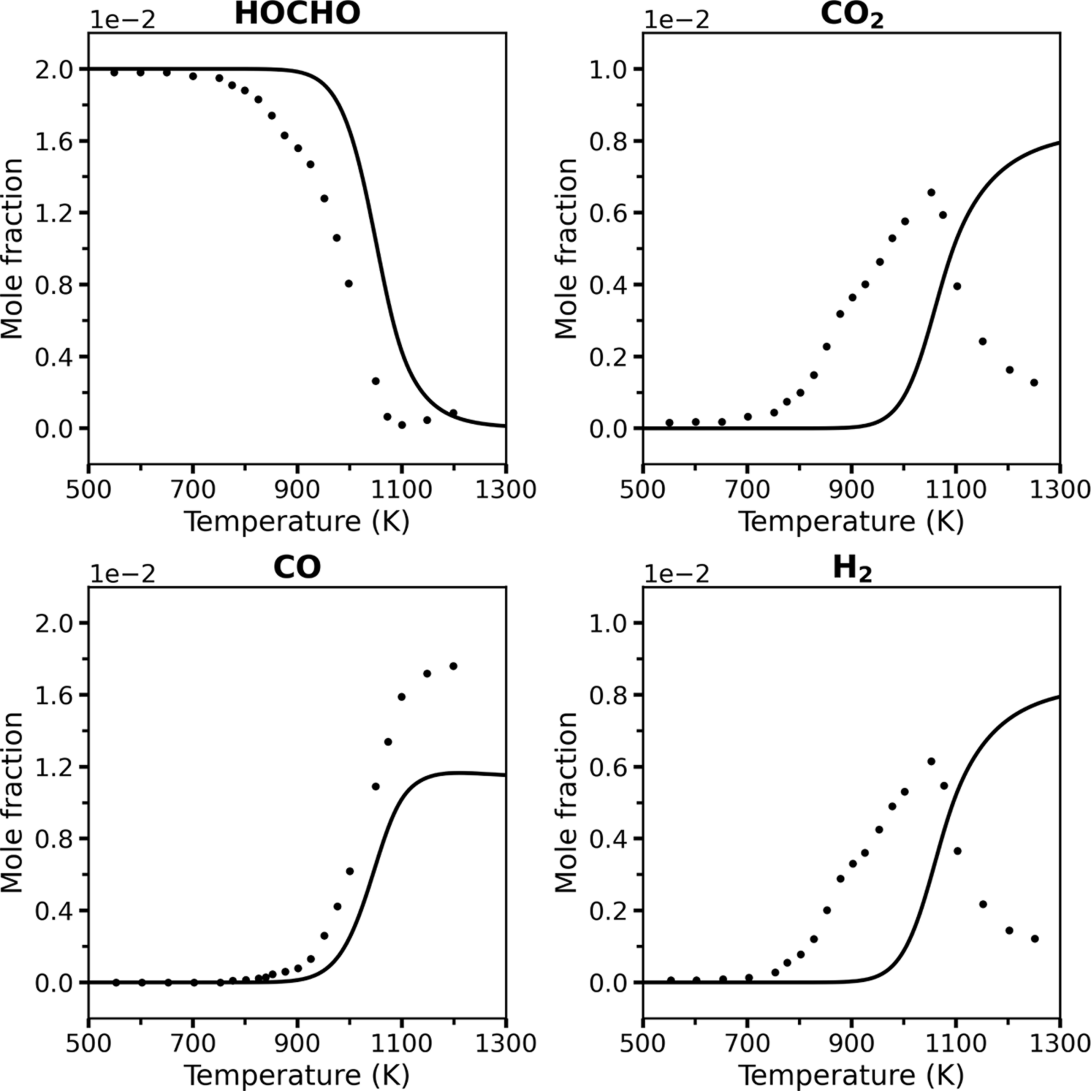
A comparison of the present “Vanilla” RMG model (solid lines) and JSR experimental data^8^ (symbols) for key species under pyrolysis conditions (2% HOCHO in He bath gas) at 1 atm, with a 2 s residence time.

The only literature model that yielded predictions with reasonable agreement with the observations for formic acid under pyrolysis conditions is the work by Yin et al.^8^ However, as discussed above, the Yin et al. model was heavily fitted to these specific conditions, making it nonpredictive and unsuitable for extension to other systems or conditions. The combination of a well-performing but nonpredictive fitted model,^8^ alongside the omission of this challenging benchmark in other key studies,^12^ has effectively masked a critical gap in the literature. As highlighted in recent perspectives in the field of predictive chemical kinetics,^24^ the tendency to overrepresent successful benchmarks while overlooking discrepancies can obscure the boundaries of current capabilities. In the case of formic acid, this reporting bias has masked a persistent challenge: the accurate prediction of pyrolysis kinetics remains unresolved for state-of-the-art (unfitted) models. By transparently documenting these limitations, rather than relying on fitted parameters to bridge the gap, we aim to set realistic expectations and identify the specific theoretical and experimental advancements required to solve this problem.

R1 $$HOCHO\left(+M\right)⇌CO+H_{2}O\left(+M\right)$$

R2 $$HOCHO\left(+M\right)⇌{CO}_{2}+H_{2}\left(+M\right)$$

The model’s failure under pyrolysis conditions points directly to an error in the predicted pressure-dependent branching ratio between the two primary decomposition channels: dehydration and decarboxylation of formic acid, Reactions R1 and R2, respectively. This is substantiated by the sensitivity analysis (Figures S5–S8), which confirms that the competition between these two channels on the CH_2_O_2_ potential energy surface (PES) exclusively controls the reactivity at pyrolysis conditions. This core deficiency in the available pressure-dependent rate coefficients for the unimolecular decomposition of formic acid shown in Figure 5 is not unique to the “Vanilla” model; the same fundamental flaw was evident in the earlier Marshall and Glarborg mechanism,^6^ as highlighted in the analysis by Yin et al.^8^ Resolving this modeling uncertainty is further complicated by the reliance on a single experimental data set for formic acid pyrolysis. New experimental studies are therefore critically needed to provide robust constraints on this branching ratio across a wider range of temperatures and pressures.

Figure 6 compares the experimentally measured and simulated laminar burning velocities (LBV, referring to the unstretched quantity, *S*_*U*_) of HOCHO/air mixtures. The “Vanilla” model reproduces the overall bell-shaped dependence of the LBV vs the equivalence ratio, with peak velocities occurring near stoichiometric conditions. At both initial temperatures, the predicted LBV values are slightly lower than the experimental values, suggesting that the model underestimates the overall reactivity of the mixture, as also noted above. Nevertheless, the shape and location of the peak are captured reasonably well, indicating that the dominant reaction pathways controlling the flame propagation are correctly represented. The remaining deviation, particularly the moderate underprediction at higher equivalence ratios, may stem from uncertainties in the rate coefficients governing the chain-branching reactions, and possibly HOCO-related reaction channels (Figure S9).

**Figure 6:**
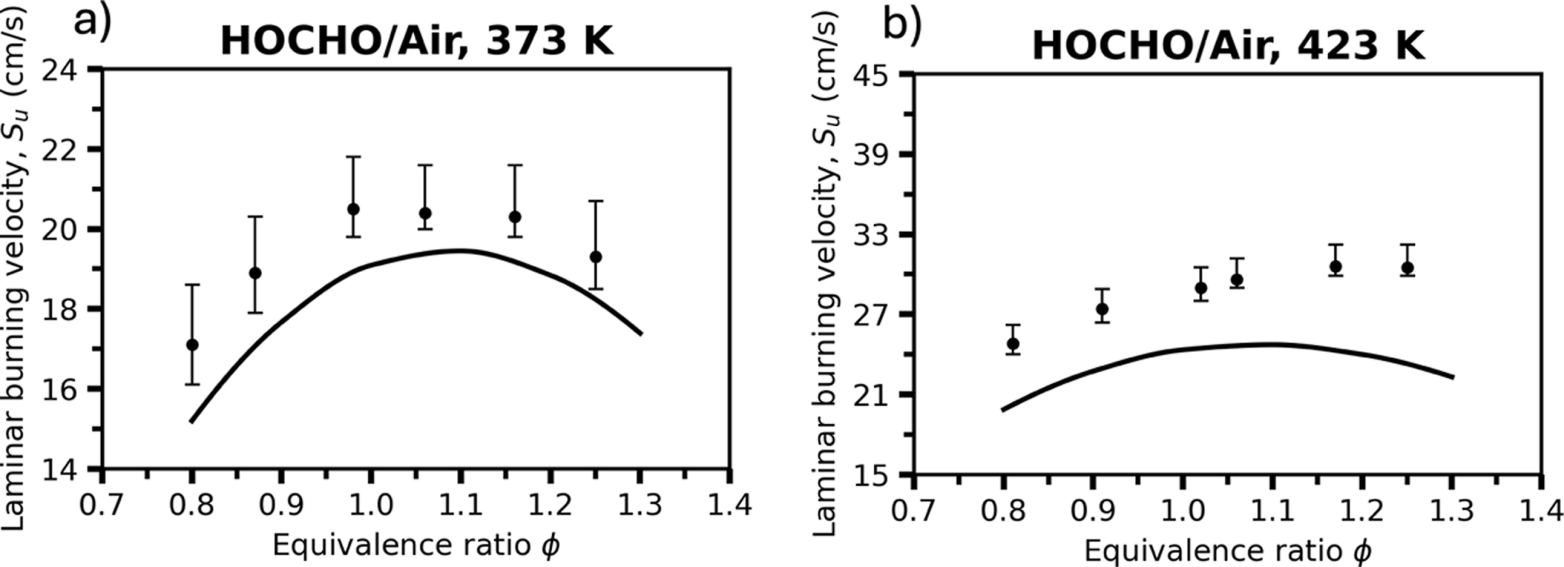
Laminar burning velocities of HOCHO/air mixtures at initial temperatures of (a) 373 K and (b) 423 K, both at 1 bar. Symbols represent experimental measurements from Sarathy et al.,^25^ while solid lines denote the present “Vanilla” RMG model results.

Figure 7 presents the measured and simulated LBV of HOCHO/O_2_/N_2_ mixtures. At 433 K, the “Vanilla” model reproduces the general trend of increasing LBV with temperature at fuel lean conditions and captures the characteristic dependence of LBV values, with the highest values approaching stoichiometric conditions. At 453 K, the experimental data display a bell-shaped profile, peaking near ϕ ≈ 1.0, while the model slightly underpredicts the magnitude of the peak but follows the overall curvature well. The observed improvement in agreement with increasing temperature indicates that the mechanism captures the enhanced radical production and chain-branching activity governing formic acid flame propagation. Discrepancies at rich conditions may arise from uncertainties in the rate coefficients of key reactions involving HOCO and CO intermediates (Figure S10).

**Figure 7:**
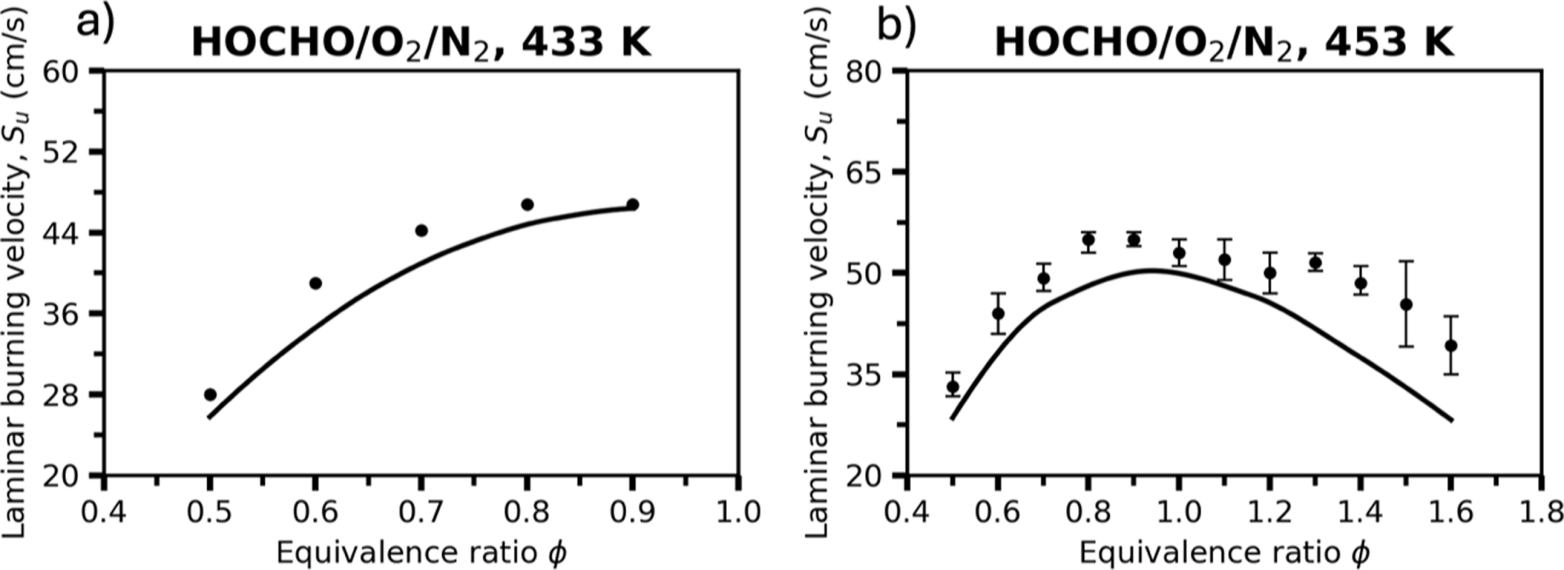
Laminar burning velocities of HOCHO/O_2_/N_2_ mixtures at initial temperatures of (a) 433 K and (b) 453 K under an oxidizer composition of 35% O_2_ and 65% N_2_, both at 1 atm. Symbols represent experimental measurements at 433 K from de Wilde and van Tiggelen^7^ (no error bars provided in the original work) and at 453 K from Yin et al.,^26^ while solid lines denote the present “Vanilla” RMG model results.

Figure 8 presents the measured and simulated LBV values of HOCHO/O_2_/Ar mixtures. Both experiment and simulation exhibit the characteristic bell-shaped dependence of the LBV values on the equivalence ratio, with the maximum LBV value occurring near ϕ ≈ 0.8–0.9. The “Vanilla” model reproduces the overall shape and magnitude of the experimental data reasonably well. Overall, the agreement confirms that the automatically generated mechanism can reliably describe the combustion behavior of formic acid even in relatively highly diluted Ar environments.

**Figure 8:**
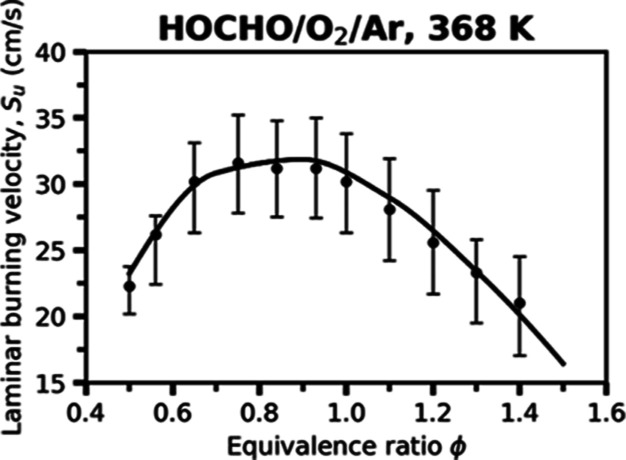
Laminar burning velocities of HOCHO/O_2_/Ar mixtures at an initial temperature of 368 K and pressure of 1 atm; the mole fraction of Ar in the oxidizer blend is 55%. Symbols represent experimental measurements from Osipova et al.,^5^ while the solid line denotes the present “Vanilla” RMG model prediction.

The strengths of the RMG software tool^13^ stem from its robust parameter estimation algorithms, which can predict thermochemical properties and rate coefficients for medium to large molecules where experimental data are often sparse. These estimation schemes, based on group additivity for thermochemistry^17^ and hierarchical family-based rate rule trees for kinetics,^14^ allow RMG to generate comprehensive mechanisms with minimal user intervention. However, for small molecules and fundamental reactions that have been extensively studied, RMG’s models achieve greater accuracy by incorporating precompiled data from curated libraries. In such cases, entries from libraries are prioritized over the estimated values, ensuring that well-known reactions are represented to the best of the modeler’s ability.

The major difference between the automated model reported previously^12^ and the current “Vanilla” RMG model is the identity and order of the libraries that were used (Table 1). Specifically, the previous automated model^12^ could have been slightly improved by adding a thermodynamic library for small H_2_/O_2_ species and a more updated C/H/O kinetic library. To aid users in navigating this crucial library selection process, the recently developed T3 framework^50,51^ now provides a systematic approach for managing and prioritizing these kinetic and thermochemical libraries automatically. It is important to note that while RMG software versions have advanced since the study by Wako et al. (v3.1.0 vs v3.3.0 used here), the core generation algorithms for C/H/O kinetics have remained methodologically consistent. The difference in model performance could perhaps be attributed to user-defined selection of thermodynamic and kinetic libraries (Table 1) and to the eventual simulation script used. However, we contend that the previous conclusion regarding the inadequacy of the automated model stemmed primarily from the truncation of the temperature range in the validation plots.^12^ For instance, Wako et al. plotted the ϕ = 1 data only up to 900 K (Figure 1), precisely the point where the unrefined RMG model begins to exhibit significant reactivity (Figure 3).

**Table 1:** Thermodynamic and Kinetic Libraries Used in Model Generation

Thermodynamic libraries^a^	
pw^b^	Wako et al.^12^
PrimaryThermoLibrary^27^	PrimaryThermoLibrary^27^
BurkeH2O2 ^28,29^	DFT_QCI_thermo^30,31^
thermo_DFT_CCSDTF12_BAC^32^	thermo_DFT_CCSDTF12_BAC^32^
DFT_QCI_thermo^30,31^	CBS_QB3_1dHR^33^
CBS_QB3_1dHR^33^	CHO^34,35^
	FFCM1(−)^36,37^

Kinetic libraries^a^	
pw^b^	Wako et al.^12^
primaryH2O2 ^38–40^	BurkeH2O2inN2 ^29,41^
2006_Joshi_OH_CO^42,43^	Klippenstein_Glarborg2016 ^44,45^
NOx2018 ^46,47^	C2H4+O_Klipp2017 ^48,49^

a Library names follow the RMG naming convention.

b pw—present work.

While pure HOCHO serves as a stringent benchmark for oxygenated-fuel kinetics, demonstrating the predictive capability for blended fuel systems is essential to establish the broader validity of the automated mechanism generation approach. To assess the generalizability of this framework to more practical combustion environments, we generated and benchmarked an additional “out-of-the-box” model for formic acid mixtures with hydrogen (H_2_) and methane (CH_4_). To capture the expanded chemical space of these cofuel systems, specifically the cross-fuel radical interactions, this generation utilized the same algorithmic parameters as the neat “Vanilla” model but was augmented with three standard RMG kinetic libraries (C2H4+O_Klipp2017, FFCM1(−), and C2H2_init). By extending the analysis to these technologically-relevant HOCHO/H_2_ and HOCHO/CH_4_ flames, we provide a rigorous test of the automated model’s robustness and its ability to capture complex fuel interactions without mixture-specific parameter tuning.

Figure 9 compares the measured and simulated laminar burning velocities for HOCHO/H_2_ and HOCHO/CH_4_ fuel blends. In the hydrogen-containing mixtures (panels a and b), the model correctly reproduces the bell-shaped dependence on the equivalence ratio and the location of the peak velocity. While a systematic modest underprediction is observed across the ϕ range, this behavior is consistent with the trend seen in neat formic acid flames (Figure 6), suggesting the deviation originates from the base fuel chemistry rather than the interactions with the added H_2_. The HOCHO/CH_4_ blends (panels c and d) exhibit excellent quantitative agreement; the simulated profiles accurately match both the magnitude and the specific shape of the experimental data, capturing the peak burning velocity near ϕ ≈ 1.05. Collectively, these results demonstrate that the extendedable, unrefined RMG model reliably captures the combustion behavior of practical formic acid blends, effectively resolving the cross-term kinetics for both hydrogen and hydrocarbon cofuels.

**Figure 9:**
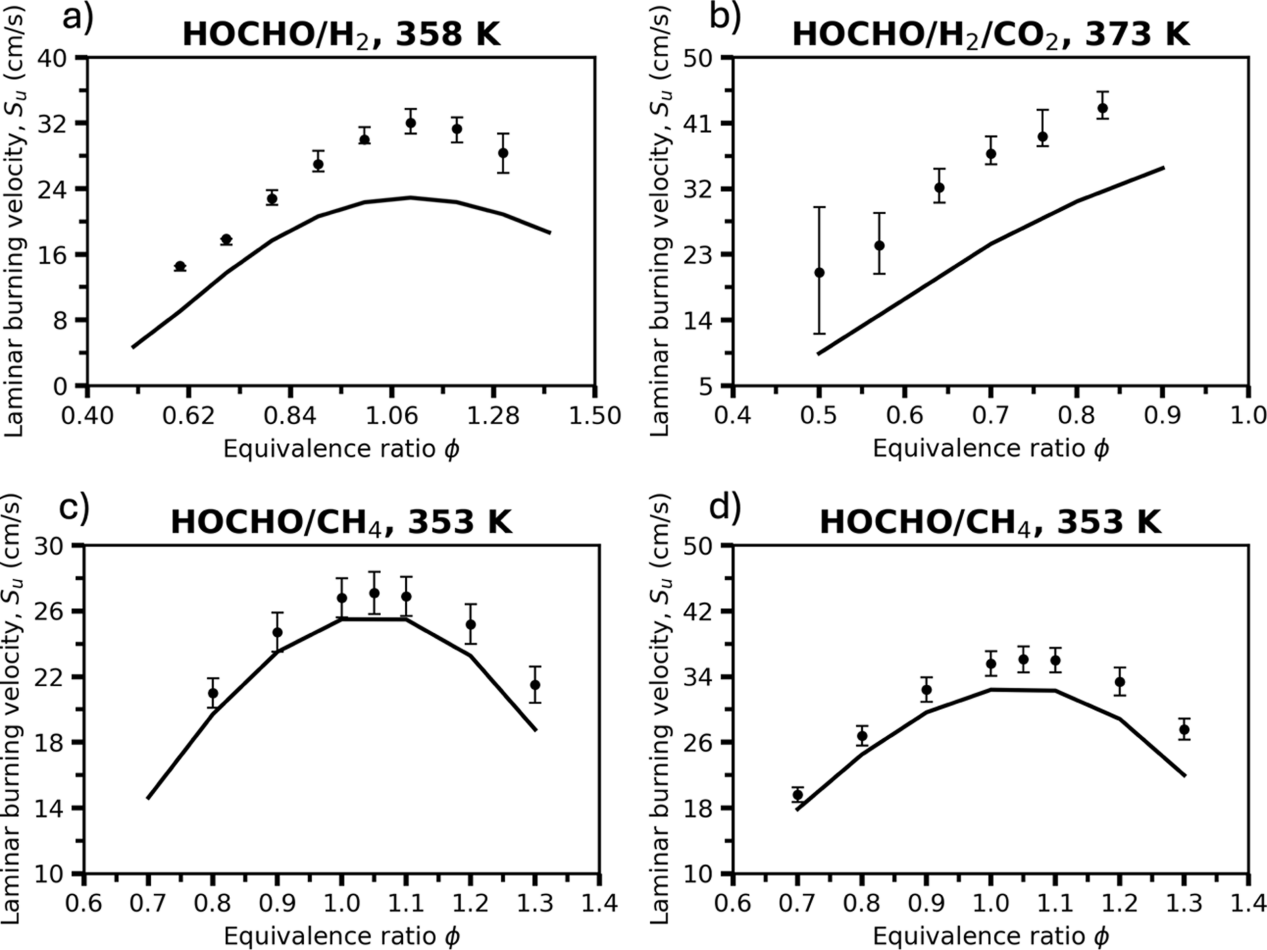
Laminar burning velocities of (a) 90% HOCHO/10% H_2_ mixtures at 358 K and (b) 25% HOCHO/37.5% H_2_/37.5% CO_2_ mixtures at 373 K, both at 1 bar, and (c) 75% HOCHO/25% CH_4_ mixtures and (d) 50% HOCHO/50% CH_4_ mixtures, both at 353 K and 1 atm. Symbols represent experimental measurements for the HOCHO/H_2_ and HOCHO/H_2_/CO_2_ systems from Sarathy et al.^25^ and for the HOCHO/CH_4_ systems from Lavadera et al.^52^ All mixtures were combusted in air. Symbols and solid lines denote the experimental observations and the present RMG model predictions, respectively. Error bars reflect the reported experimental uncertainties.

Quantifying curated vs automatically generated kinetics in the “Vanilla” formic acid/methane/hydrogen mechanism shows that of 2992 total reactions, ∼7% were sourced from kinetic libraries, ensuring the foundational chemistry was anchored by high-quality literature values. The remaining reactions, including template-based estimations (∼37%) and pressure-dependent reactions (∼56%), were generated automatically by RMG’s algorithms. This distribution illustrates that while library selection is critical for the core, the majority of the reaction network arises from RMG’s predictive rules.

Figure 10 presents detailed reaction-pathway flux diagrams for formic acid in a JSR at 1200 K, 1 atm, and 2 s residence time. The dominant decomposition channels under pyrolysis conditions (Figure 10A) are the dehydration and decarboxylation pathways, Reactions R1 and R2, shown by the thickest arrows. Both routes proceed directly to stable products, and only a minor fraction (<1%) passes through HOCO, indicating limited radical propagation in the absence of an oxygenating environment. No multistep branching sequences are observed, reflecting the characteristic simplicity of formic acid pyrolysis chemistry.

**Figure 10:**
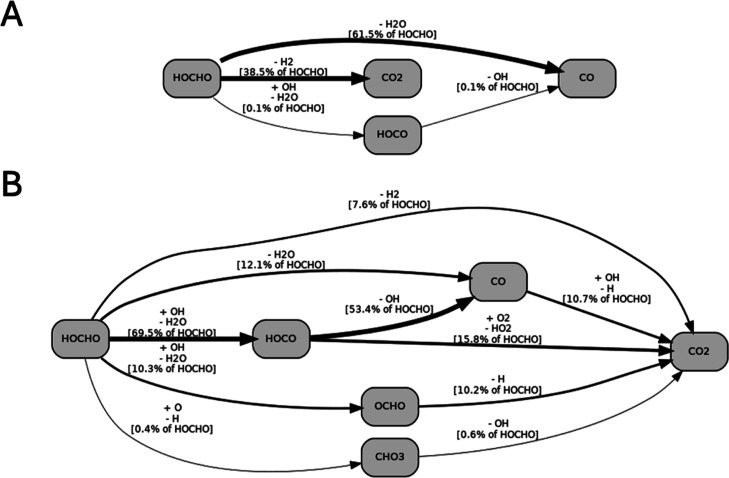
Flux diagrams of formic acid decomposition at 1200 K, 1 atm, and 2 s in a JSR, automatically generated by T3.^50^ (A) Pyrolysis of 2% formic acid in an inert bath gas. (B) Oxidation of formic acid in air, ϕ = 0.5. Arrow widths correspond logarithmically to the respective rate of production.

Under oxidation conditions (Figure 10B), dehydration and decarboxylation remain relatively significant, but radical-chain propagation, primarily via OH attack, becomes prominent. The HOCHO → HOCO channel becomes the dominant entry point into the reactive pool, accounting for ∼70% of total HOCHO consumption at the examined conditions. Dehydration contributes ∼12%, while the HOCHO → OCHO route (∼10%) exceeds the direct decarboxylation channel flux, indicating OCHO formation of a more significant intermediate under oxidizing conditions than a direct CO_2_ + H_2_ split. HOCO subsequently branches along two primary directions: a major flux toward CO and a smaller, oxygen-assisted route toward CO_2_. Minor fluxes continue through OCHO and CHO_3_, which feed back into the CO_2_ pool through radical-mediated oxidation sequences. Unlike pyrolysis, CO is not a terminal product; it oxidizes into CO_2_ primarily through CO + OH → CO_2_ + H. CO_2_ is indeed the dominant carbon sink at oxydizing conditions.

## 4 Conclusions

This work demonstrates that a predictive, “out-of-the-box” chemical kinetic model (i.e., without quantum chemical refinement) for formic acid, built automatically with RMG, can achieve performance on par with and, in some respects, superior to existing literature mechanisms. The primary goal was not to claim the “best” formic acid model but rather to advance the field by revisiting and correcting two prevailing misconceptions that have hindered progress, transparently showing model failures alongside successes.

The perception that formic acid kinetics are effectively “solved” illustrates a broader community challenge: the tendency to emphasize successful predictions while minimizing attention to systematic errors or fundamental gaps. In the case of formic acid, this misconception stemmed from two sources: the juxtaposition of a rigorous PES analysis with excellent validation results achieved via simplified parameter fitting, which created a potential for misinterpretation regarding the model’s physical fidelity (a commission),^8^ and not benchmarking models against available and more challenging data (an omission).^12^ Together, these previous studies suggest that the pressure-dependent branching ratio of formic acid decomposition is fundamentally resolved, whereas the agreement was actually derived from manual adjustments. These practices masked the central, unresolved scientific challenge: the pressure-dependent branching ratio in the CH_2_O_2_ PES.

The second misconception is that automated mechanisms underperform manual versions. Previous work suggested that automated generation yields negligible reactivity for this system, implying that extensive parameter fitting is unavoidable.^12^ The present work clearly shows that an unrefined and unfitted predictive chemical kinetic model performs adequately well in predicting formic acid speciation data vs temperature at different equivalence ratios and laminar burning velocities vs equivalence ratio at different compositions.

By correcting these views, the present work reveals the true state-of-the-art and provides a clear direction for improvement. The key scientific hurdle for any future formic acid model is the accurate parameterization of the pressure-dependent kinetics on the CH_2_O_2_ PES. Specifically, obtaining accurate phenomenological rate coefficients that capture the competition between the decarboxylation and dehydration channels is essential for reliable predictions of formic acid reactivity. Resolving this modeling uncertainty is further complicated by the reliance on a single experimental data set for formic acid pyrolysis. New experimental studies are therefore critically needed to provide robust constraints on this pressure-dependent branching ratio across a wider range of temperatures and pressures.

The “Vanilla” RMG model thus serves as a new transparent benchmark: a truly predictive first-iteration mechanism that requires no ad hoc fitting. It achieves reasonable agreement where the chemistry is well-defined while honestly exposing the specific areas that require further fundamental study. While parameter fitting is often necessary to resolve the remaining uncertainties for engineering applications, it should follow, not precede, the establishment of a physically sound reaction network. In this case, premature fitting in the literature masked the structural deficiency of the model regarding the CH_2_O_2_ pressure-dependent branching ratio. By prioritizing physical fidelity over perfect agreement, our approach exposes this underlying scientific challenge rather than obscuring it, emphasizing the importance of transparency. Sharing negative results and out-of-domain discrepancies alongside successes is essential to define the current boundaries of predictive capability, thereby transparently advancing science. To genuinely advance the predictive chemical kinetics field, validation should be performed against all meaningful available data, not a cherry-picked subset, and unjustified parameter fitting should be avoided in favor of a mechanistic understanding. This transparent, challenge-driven approach is essential for constructive community feedback and progress.

## Supplementary Material





## Notes

A PDF file with sensitivity analysis results for JSR pyrolysis and oxidation condition and LBV simulations (PDF)
A text file with the RMG input that was used for automated model generation, a text file with the RMG model in Chemkin format, a text file with the RMG model in Cantera format, and a text file with the Cantera simulation script (ZIP)
