# Supplementary material for: Using RMG Out-of-the-Box for Formic Acid Pyrolysis and Oxidation
Source: ACS Omega. 2026 Jan 15;11(4):6371–81. doi: 10.1021/acsomega.5c11182 (PMC12878340; doi:10.1021/acsomega.5c11182)
Supplement: Supplementary file 1 [file ao5c11182_si_001.pdf]

Supporting Information for the paper:

Using RMG Out-of-the-Box for Formic Acid Pyrolysis and Oxidation

Jintao Wu,[a] and Alon Grinberg Dana[a,b,*]

[a]Grand Technion Energy Program (GTEP), Technion – Israel Institute of Technology, Haifa 3200003, Israel
[b]Wolfson Department of Chemical Engineering, Technion – Israel Institute of Technology, Haifa 3200003, Israel

* Corresponding author, alon@technion.ac.il

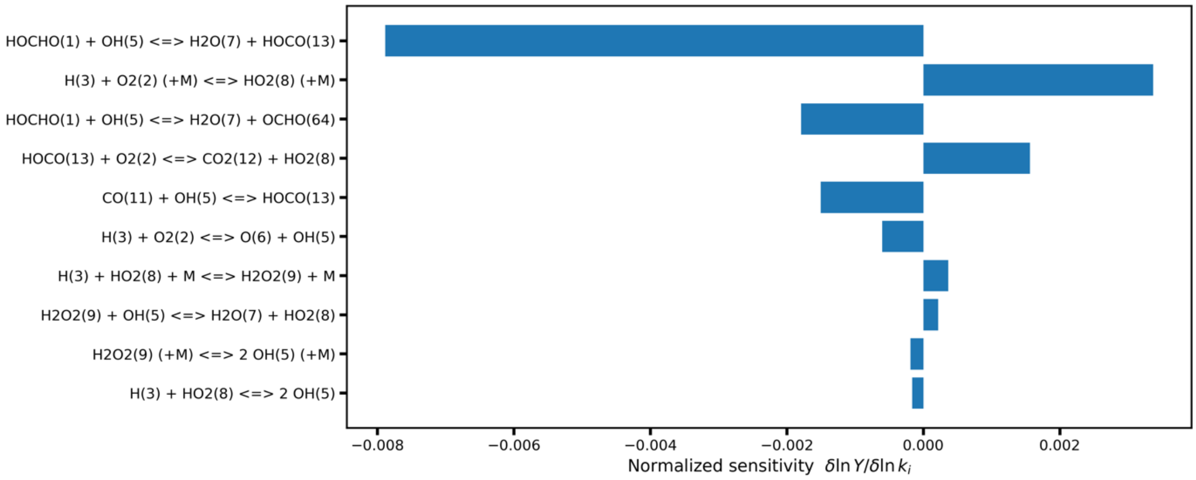

Figure S1: Sensitivity analysis at oxidation condition $\phi = 0.5$ of HOCHO as the observable, T=1100K, t=2.0 s.

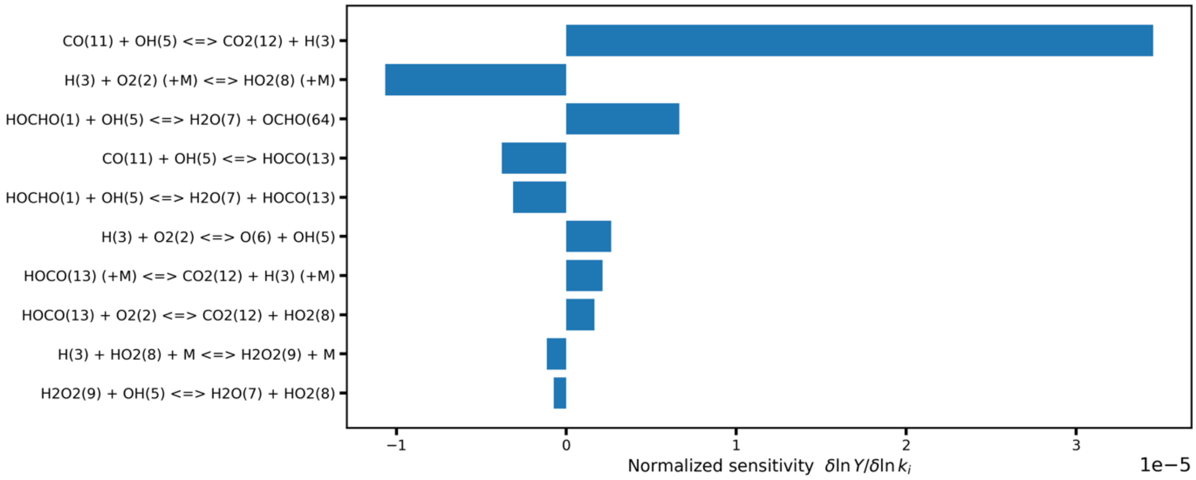

Figure S2: Sensitivity analysis at oxidation condition $\phi = 0.5$ of CO_2 as the observable, T=1100K, t=2.0 s.

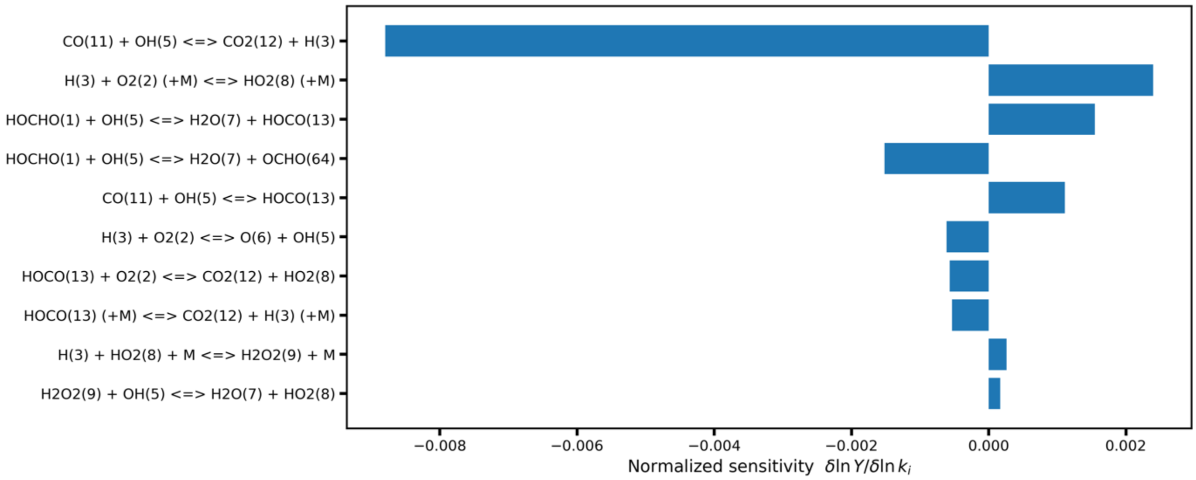

Figure S3: Sensitivity analysis at oxidation condition $\phi = 0.5$ of CO as the observable, T=1100K, t=2.0 s.

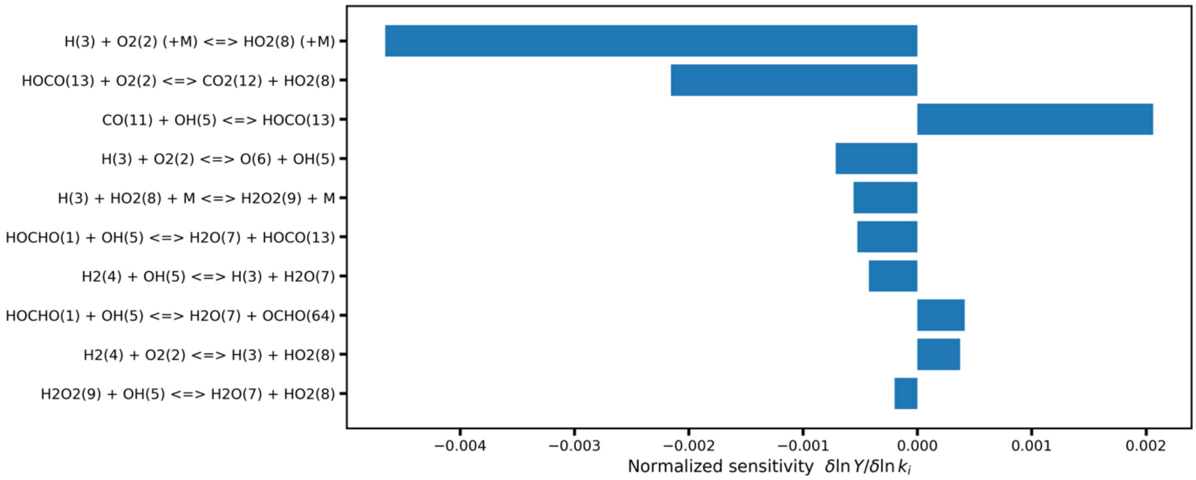

Figure S4: Sensitivity analysis at oxidation condition $\phi = 0.5$ of H_2 as the observable, T=1100K, t=2.0 s.

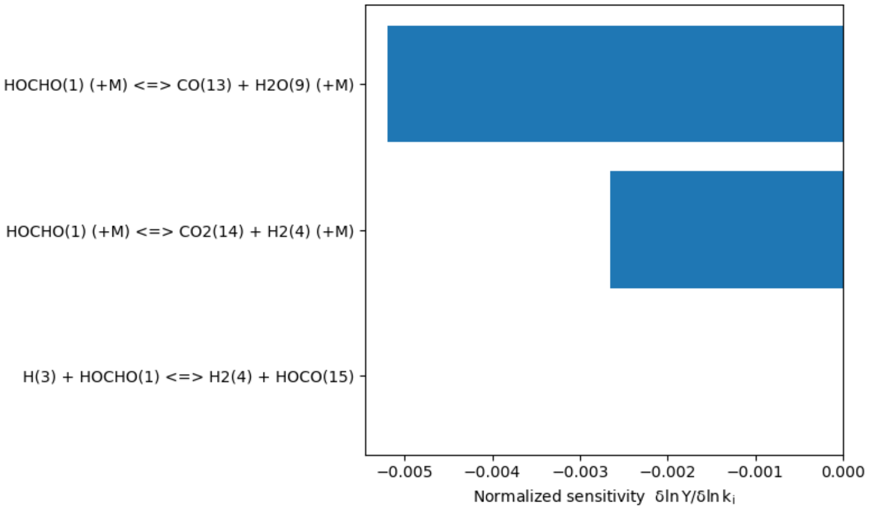

Figure S5: Sensitivity analysis at pyrolysis condition of HOCHO as the observable, T=1100K, t=2.0 s.

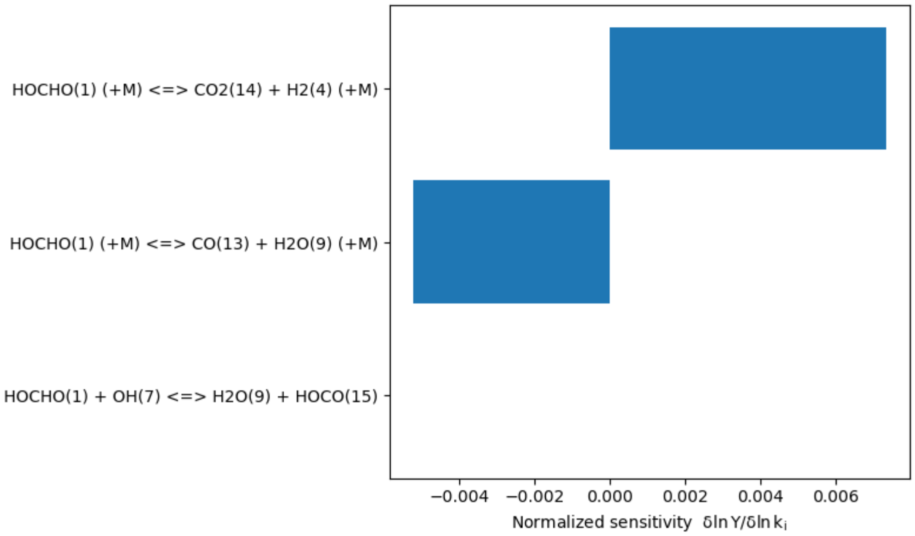

Figure S6: Sensitivity analysis at pyrolysis condition of CO_2 as the observable, T=1100 K, t=2.0 s.

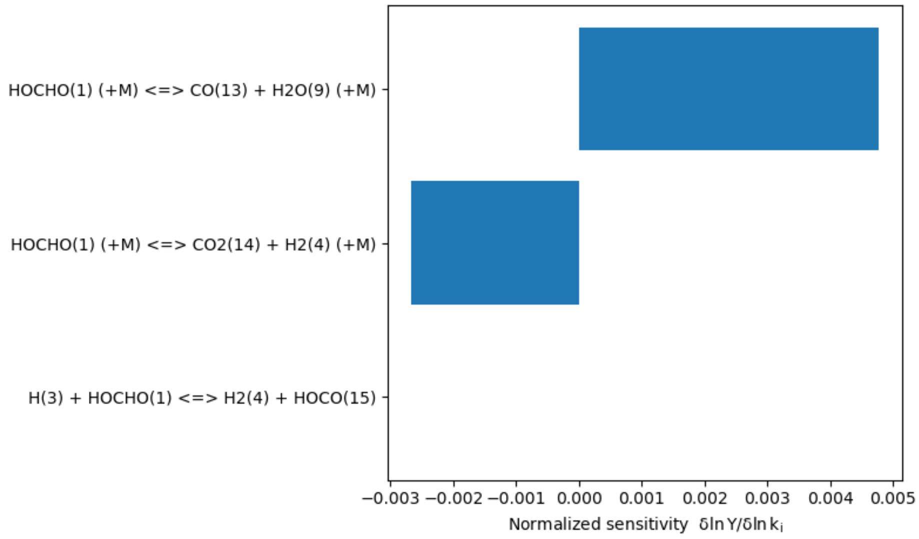

Figure S7: Sensitivity analysis at pyrolysis condition of CO as the observable, T=1100 K, t=2.0 s.

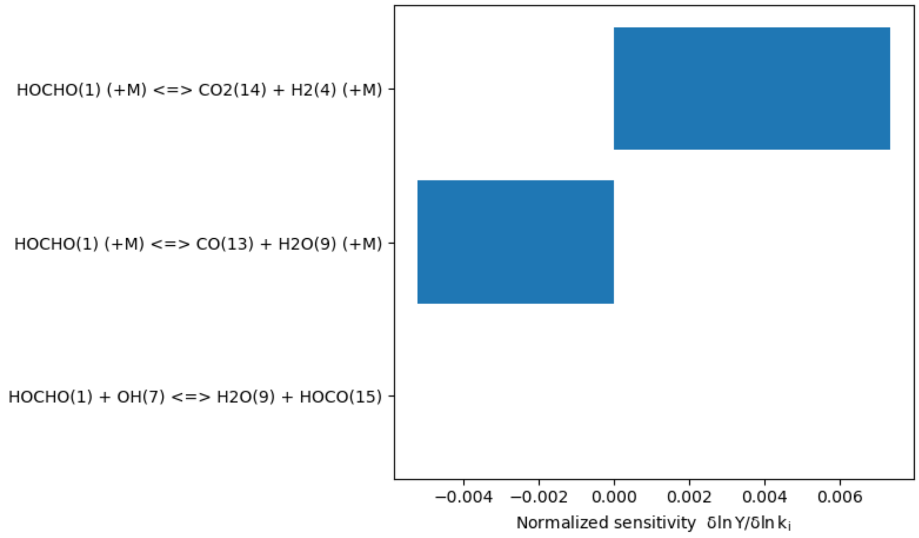

Figure S8: Sensitivity analysis at pyrolysis condition of H_2 as the observable, T=1100 K, t=2.0 s.

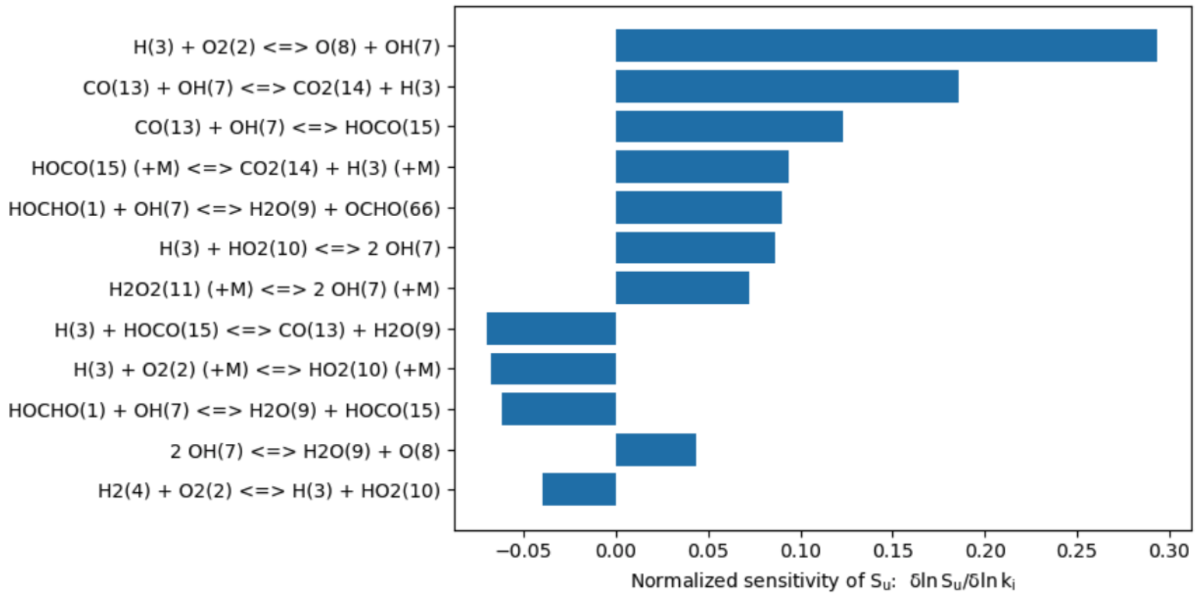

Figure S9: Sensitivity analysis of the relative influence of individual reactions on the laminar burning velocity for HOCHO/air mixtures at initial temperatures of 423 K and 1 bar, φ=1.00, Su=24.54 cm/s.

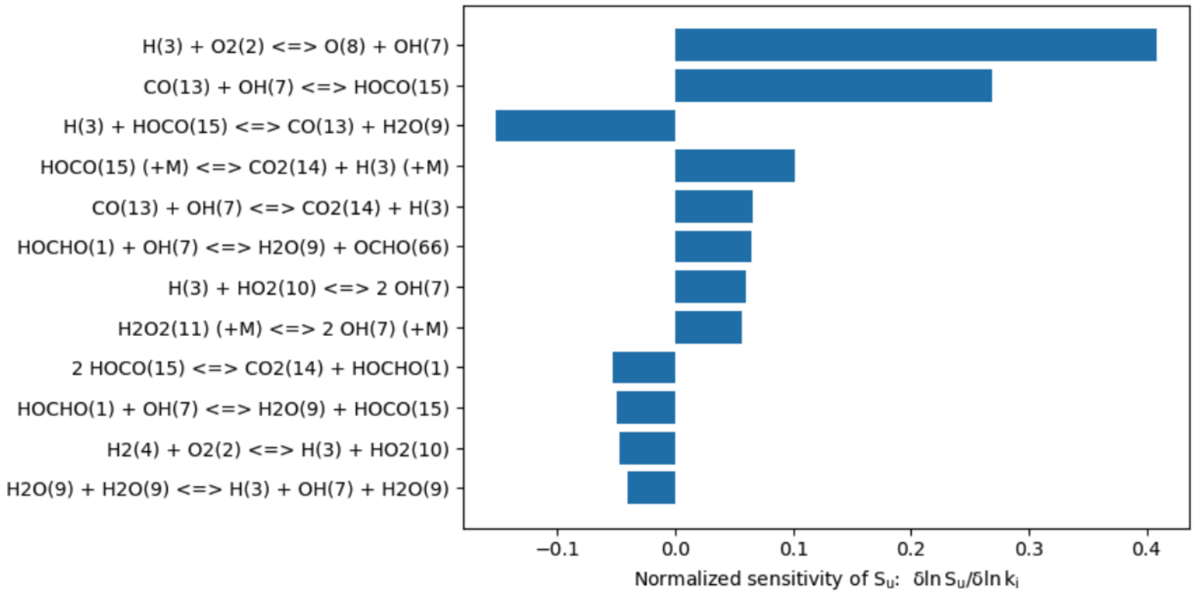

Figure S10: Sensitivity analysis of the relative influence of individual reactions on the laminar burning velocity for HOCHO/O_2/N_2 mixtures at initial temperatures of 453 K under an oxidizer composition of 35% O_2 and 65% N_2, both at 1 atm, φ=1.60, Su=28.22 cm/s.

Table S1: A comparison of software versions and RMG input parameters between the automatically generated model in literature[1] and in the present work.

**Parameter**	**Wako et al. (2022)[1]**	**Current Work ("Vanilla")**
RMG Software Version	v 3.1.0	v 3.3.0
Flux Tolerance (toleranceMoveToCore)	0.012	0.001
Edge Flux Tolerance (toleranceInterruptSimulation)	0.02	0.001<br>(should be equal to toleranceInterruptSimulation unless using memory pruning)
Edge Memory Pruning (toleranceKeepInEdge)	0.01	0.0 (default)
Temperature Range	400 – 2000 K	450 – 2000 K
Pressure Range	1 – 100 bar	1 – 50 bar
Termination Criteria	The earliest between 50 s and a 99% fuel conversion	5 s

References:

[1] Wako, F. M.; Pio, G.; Salzano, E. Modeling Formic Acid Combustion. Energy & Fuels 2022, 36, 14382.
